# High-Density Lipoproteins as Homeostatic Nanoparticles of Blood Plasma

**DOI:** 10.3390/ijms21228737

**Published:** 2020-11-19

**Authors:** Vasily A. Kudinov, Olga Yu. Alekseeva, Tatiana I. Torkhovskaya, Konstantin K. Baskaev, Rafael I. Artyushev, Irina N. Saburina, Sergey S. Markin

**Affiliations:** 1 Laboratory of Cell Biology and Developmental Pathology, FSBSI Institute of General Pathology and Pathophysiology, 125315 Moscow, Russia; saburina@mail.ru; 2 Experimental Drug Research and Production Department, Institute of Biomedical Chemistry, 119121 Moscow, Russia; konstantinbaskaev@gmail.com (K.K.B.); rifiraf21ibmx@gmail.com (R.I.A.); 3 Cell Physiology Laboratory, Institute of Biomedical Problems, Russian Academy of Sciences, 123007 Moscow, Russia; aou_13@mail.ru; 4 Department of Biochemistry, People’s Friendship University (RUDN University), 117198 Moscow, Russia; 5 Laboratory of Phospholipid Transport Systems and Nanomedicines, Institute of Biomedical Chemistry, 119121 Moscow, Russia; torti@mail.ru; 6 Clinical Research Department, Institute of Biomedical Chemistry, 119121 Moscow, Russia; comphos@mail.ru

**Keywords:** high-density lipoproteins, HDL functions, reverse cholesterol transport

## Abstract

It is well known that blood lipoproteins (LPs) are multimolecular complexes of lipids and proteins that play a crucial role in lipid transport. High-density lipoproteins (HDL) are a class of blood plasma LPs that mediate reverse cholesterol transport (RCT)—cholesterol transport from the peripheral tissues to the liver. Due to this ability to promote cholesterol uptake from cell membranes, HDL possess antiatherogenic properties. This function was first observed at the end of the 1970s to the beginning of the 1980s, resulting in high interest in this class of LPs. It was shown that HDL are the prevalent class of LPs in several types of living organisms (from fishes to monkeys) with high resistance to atherosclerosis and cardiovascular disorders. Lately, understanding of the mechanisms of the antiatherogenic properties of HDL has significantly expanded. Besides the contribution to RCT, HDL have been shown to modulate inflammatory processes, blood clotting, and vasomotor responses. These particles also possess antioxidant properties and contribute to immune reactions and intercellular signaling. Herein, we review data on the structure and mechanisms of the pleiotropic biological functions of HDL from the point of view of their evolutionary role and complex dynamic nature.

## 1. Introduction

Lipoproteins were initially defined according to their composition (i.e., lipids and proteins) and classified according to their density (from very low- to high-density lipoproteins (HDL)). Historically, the most interest in HDL was linked to the negative correlation between HDL cholesterol (HDL-C) level and risk of coronary artery disease (CAD), as established by the Framingham study, concluding that low HDL-C levels are as much a risk factor for CAD as high low-density lipoprotein cholesterol (LDL-C) levels [1]. These data were later confirmed by other studies [2,3], and it is believed that they can be explained by the role of HDL in reverse cholesterol transport (RCT) from the macrophages in peripheral tissues to the liver for disposal [4].

Although it was originally believed that the level of HDL-C was the most important determinant of these processes, HDL-C-raising therapies all failed to improve cardiovascular outcomes. Clinical trials of cholesteryl ester transfer protein (CETP) inhibitors were very disappointing and ended with a conclusion of “insufficient cardiovascular benefit for routine use” [5]. Indeed, emerging evidence indicates that the composition and, consequently, the function of HDL is perhaps the most important factor, rather than their quantity and that the HDL-C concentration reflects only one side of the story. Dysfunctional HDL are unable to protect blood vessels, and, therefore, new strategies to measure and restore HDL functionality are needed [6].

Nowadays, it is widely accepted that HDL are a class of natural nanoparticles that display pleiotropic functions, such as RCT, anti-inflammatory action, immunity, antioxidative effects, and antithrombotic action [7,8,9,10]. Proteomics, lipidomics, and the analysis of small RNA in HDL particles are uncovering more and more of the functions of HDL. For instance, it has been established that HDL are complex particles that undergo dynamic remodeling through interactions with various enzymes and tissues throughout their life cycle, making the complete understanding of their functions and roles more complicated than initially expected [11].

The great interest in HDL pertains to the understanding of their functions from an evolutionary point of view, which shows that these ancient blood particles are not only lipid transporters but also display important functions in many aspects of immunity, tissue homeostasis, and intercellular signaling in both invertebrates and vertebrates. The protective effects of HDL in cardiovascular disease rely on this broad spectrum of homeostatic properties.

It seems that the time has come for us to leave our canonical views of HDL as lipid transporters and to crystallize the new data on HDL pleiotropic functions in favor of a new concept of HDL as an ancient dynamic platform for maintaining many aspects of an organism’s homeostasis. It is obvious that some of the protective properties of HDL can be used to improve the treatment of many acute and chronic diseases and to develop new HDL-based medicines.

In this review, we summarize the data on the dynamic structure and pleiotropic functions of HDL to update the information about them and highlight the potential for the therapeutic use of HDL’ functionality as a prospective biomimetic therapeutic platform.

## 2. Composition and Structure of HDL

“HDL” were discovered in 1929 when a lipid-rich α-globulin was isolated from horse serum by Macheboeuf at the Pasteur Institute in Paris [12]. HDL are a family of particles that can exhibit fundamentally different metabolisms and functions based on their specific proteomic, lipidomic, and physicochemical properties. HDL are the smallest lipoproteins (LPs) of blood serum with a density of 1.063–1.210 g/mL and a particle size of 7–12 nm, and, according to modern terminology, they are ultrasmall natural nanoparticles. The structure of HDL is similar to all LPs: HDL consist of the hydrophobic nucleus of non-polar lipids (triglycerides and esterified cholesterol) surrounded by a monolayer of phospholipids and free cholesterol, with inclusions of protein molecules named apoproteins. HDL are a heterogeneous fraction of LPs with several subfractions of various densities, sizes, forms, electrophoretic mobilities, protein–lipid compositions, and physiological functions [13,14]. The first method of separation of blood plasma LPs to fractions was electrophoresis in agarose gel. HDL are characterized by higher mobility compared to other LPs and thus produce a separate band marked as α-LPs. The band of α-LP fractions differs from other bands of larger and lighter LPs of other classes, such as β- and preβ-LPs.

The wide heterogeneity of HDL fractions—the difference in the ratio of lipid and protein components—and the resulting differences in the densities and sizes (diameter) of the particles determine the differences in the principle of their classification. According to Jomard and Osto [11], the most convenient for clinical usage is nuclear magnetic resonance (NMR)-based HDL classification [15], identifying five distinct HDL subfractions [16]: very large HDL particles, large HDL particles, medium HDL particles, small HDL particles, and very small HDL particles. This nomenclature also includes an entry for the pre-β-1 HDL subclass that participates in macrophage cholesterol efflux [15]. Despite the application of this approach in a number of works [17], in most studies, the same fractions were separated by ultracentrifugation, based on differences in their densities, and, similar to other plasma LPs, the density of HDL molecules serves as the main criterion of their classification. Ultracentrifugation is also the main method for the separation of α-LP subfractions. Based on the differences in particle densities, HDL_2_ and HDL_3_ fractions with densities of 1.063–1.125 and 1.125–1.21 g/mL, respectively, have been isolated. In a number of works, along with this, deeper fractionation of each of these two fractions has been carried out. In accordance with the results of electrophoretic separation of HDL subfractions in gradient non-denaturing polyacrylamide gel electrophoresis (GGE), they can be separated into subfractions with different molecule sizes. Two HDL_2_ and three HDL_3_ subclasses have been identified and their particle size characterized by this method: HDL_3c_, 7.2–7.8 nm diameter; HDL_3b_, 7.8–8.2 nm; HDL_3a_, 8.2–8.8 nm; HDL_2a_, 8.8.–9.7 nm; and HDL_2b_, 9.7–12.0 nm [18] (Table 1).

The division of HDL into separate big (HDL_2_), medium (HDL_3a_), and small (HDL_3b_ + HDL_3c_) subfractions by particle size has been confirmed by NMR-spectroscopy [19] and matches the above-proposed classification of five subfractions based on size [11]. Moreover, the additional LP fraction, also considered as a class of HDL, has been revealed in plasma fractions with a density of more than 1.21 g/mL. These LPs are pre-beta-migrating when separated by agarose gel electrophoresis and are called preβ-HDL. Apoprotein A-I (ApoA-I), which is present in all HDL fractions, is the main component of this subfraction [20], which is lipid-poor and gradually enriched by lipids in circulation.

Preβ-HDL are synthesized in the liver and small intestine. They consist of ApoA-I, phospholipids, and free cholesterol released from cells. Due to the absence of non-polar lipids in their structure, preβ-HDL form disk-shaped structures, called newly developed or nascent HDL [21]. Nascent HDL are quickly transformed into spherical HDL during the circulation and esterification of cholesterol. Cholesterol esterification is catalyzed by lecithin–cholesterol acyltransferase (LCAT), and disk-shaped nascent HDL may be revealed in plasma only in familial LCAT deficiency. Cholesterol esters generated during this reaction form the hydrophobic nucleus of HDL particles. This process is accompanied by the production of small HDL_3_ subfractions following the transformation of these particles [22,23].

The high dynamics of HDL subfractions in blood serum, a constant exchange of lipid and protein compounds, and the transformation of fractions into one another during HDL remodeling should be emphasized. In fact, the boundaries of the densities and sizes of HDL subfractions given in Table 1 are somewhat arbitrary, and there are no clear boundaries between them, unlike other, lighter classes of LPs. In the process of the circulation of the smallest particles, starting from HDL_3_ and even earlier (preβ-HDL), they are gradually enriched with lighter (compared to protein) lipid components, which can also be exchanged with other blood components while increasing in size. The density of the particles is gradually reduced, and HDL turn into larger and lighter HDL_2b_. The ratio between different HDL subfractions indirectly reflects the intensity of these transformation processes, called remodeling [24]. The information about HDL remodeling remains incomplete, but it has been shown that this process is related to certain functions of these LPs. The analysis of HDL by modern proteomic mass spectrometry methods has allowed the identification of several short-living subfractions formed due to the temporary association of HDL particles with certain plasma proteins among the abovementioned fractions [25].

The content of protein molecules in HDL is approximately 50% of the particle weight. More than 90% of HDL proteins are composed of ApoA-I and ApoA-II proteins; the ratio of ApoA-I and ApoA-II in the HDL_2_ and HDL_3_ is approximately 3:1, while ApoC composes only 3%–5% of the proteins of HDL_2_ and 1%–2% of the proteins of HDL_3_. However, approximately half of ApoC observed in blood plasma under fasting conditions is a part of HDL. HDL are considered a depot of ApoC, which is released from chylomicrons and very-low-density lipoproteins (VLDL) during lipolysis [26]. Besides the abovementioned apolipoproteins, HDL contain other minor newly observed apoproteins, as well as an enzyme paraoxonase [27].

Several enzymes and transport proteins mediating lipid metabolism, which are bound to HDL, are present in plasma. The association of these molecules and HDL has been shown by mass spectrometry-based proteomics with high sensitivity. HDL fractions contain up to 80 of these proteins [27]. The main proteins permanently or temporarily bound to HDL, as well as their properties and functions, are shown in Table 2.

The multiple effects of apoproteins ApoF, -H, -J, -L, and -M, despite their low content in HDL, reflect their high biological activity and influence on HDL functions [28]. It might also suggest that, besides the effects of the apoproteins listed in Table 2, some of these agents or their combinations might contribute to the maintenance of the surface structure of HDL. These apoproteins might also determine the temporary association of various functional proteins circulating in plasma with HDL particles, which are mentioned in Table 3.

The high number of associated HDL proteins, along with the wide spectrum of functions, determine the multifunctionality of HDL and their contribution to various biological processes. For example, the level of the serum amyloid protein SAA1 in HDL negatively correlates with the ability to uptake cholesterol [29]. Modifications of HDL proteins, particularly the main protein ApoA-I, are observed under certain conditions. In particular, oxidized methionine in the 148 position of this protein has been shown in patients with cardiovascular disorders and type II diabetes. The observed impairments might be associated with the dysfunction of HDL [30,31]. Proteomic analysis of HDL in vitro exposed to acrolein (an aldehyde of cigarette smoke) has shown the appearance of cross-links between apoproteins and acrolein adducts of ApoA-I and ApoA-II. The authors proposed that these changes might contribute to the atherogenic effects of smoking by the reduction in HDL activity [32].

## 3. Functions of HDL

The further functions of HDL in organisms are discussed in this section in detail.

### 3.1. Reverse Cholesterol Transport (RCT)

RCT, the transport of excess cholesterol from the peripheral tissues to the liver for utilization and excretion with the bile [33], is considered a classical function of HDL. The term RCT was first proposed in 1968 [34], and in accordance with Glomset’s hypothesis, excess free cholesterol is toxic for cells. Cells of peripheral tissues cannot excrete excess cholesterol, and its accumulation in the endoplasmic reticulum (EPR) might result in protein folding impairments, thus triggering apoptosis. To prevent these processes, excess cholesterol should be removed from the cells by RCT [20]. The main stages of RCT were originally described by Havel and Small in 1987 [35,36] and then later investigated [37,38,39,40,41], and are as follows:

- Cholesterol mobilization and transport to the plasmatic membrane of the cell.
- Transport of free cholesterol from the plasmatic membrane to acceptor particles (HDL) by the participation of membrane-localized ATP-binding cassette transporters ABCA1 and ABCG1 [37]. A significant role in this process belongs to the main phospholipid (PL) of HDL, phosphatidylcholine [42].
- Esterification of free cholesterol bound to HDL by LCAT.
- Transport of cholesterol esters to hepatocytes by mature HDL.
- Engulfment of cholesterol esters from mature HDL particles mediated by the hepatocyte scavenger receptor class B type I (SR-BI) [38].
- Transport of cholesterol esters to hepatocytes by mature low-density lipoproteins (LDLs) due to cholesterol exchange to HDL mediated by CETP.

**Figure 1:**
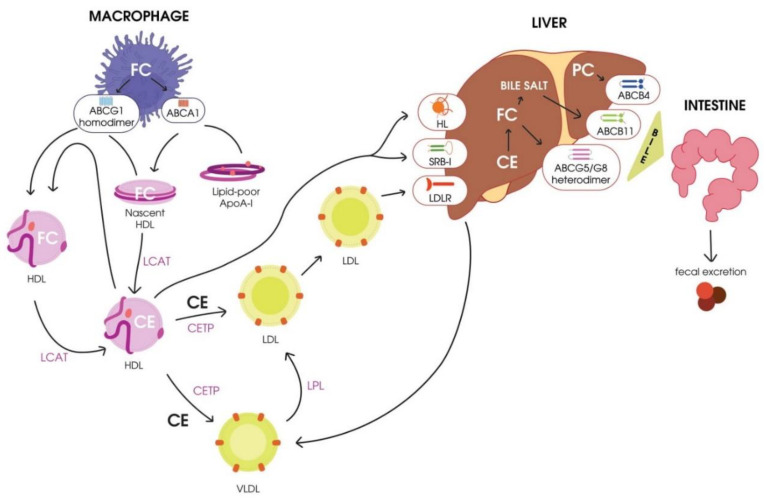
Schematic representation of reverse cholesterol transport. HDL, high-density lipoprotein; CETP, cholesterol ester transport protein; LCAT, lecithin–cholesterol acyltransferase; FC, free cholesterol; CE, cholesteryl ester; HL, hepatic lipase; VLDL, very-low-density lipoprotein; LPL, lipoprotein lipase; LDL, low-density lipoprotein; LDLR, LDL receptor; PC, phosphatidylcholine; SR-BI, scavenger receptor class B type I.

A scheme of RCT is presented in Figure 1.

### 3.2. Non-Classical Functions of HDL

**Figure 2:**
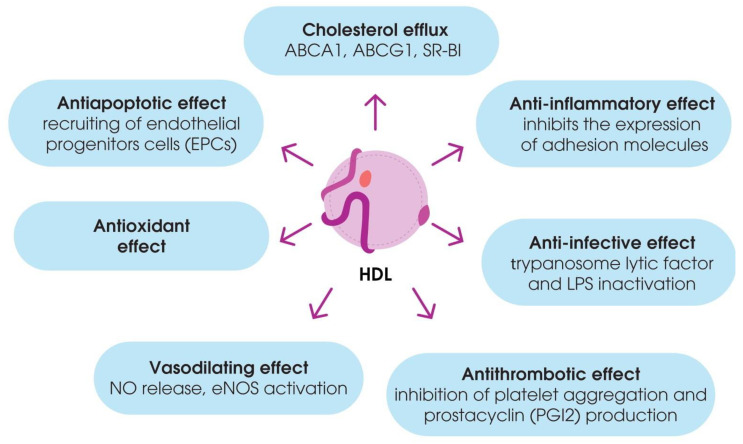
Pleiotropic effects of high-density lipoproteins (HDL).

Besides their contribution to RCT, HDL have been shown to have antioxidant properties to modulate inflammatory responses, vasomotor reactions, and blood clotting, as well as to mediate immune responses. These functions of HDL are often impaired during dyslipidemia and atherosclerosis [43] (Figure 2).

The various pleiotropic effects of HDL are briefly presented below.

#### 3.2.1. Immune Reactions

**Figure 3:**
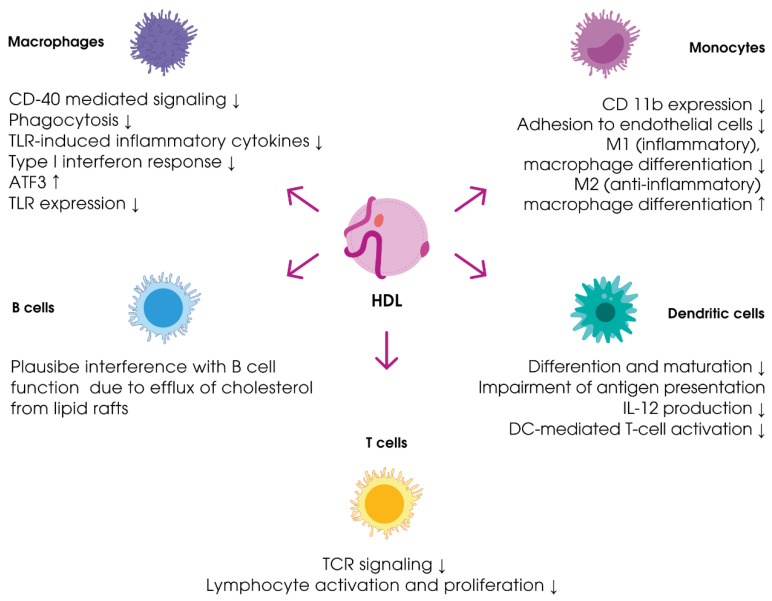
Effects of HDL on cells of the immune system (adapted from [44]). ATF3, activating transcription factor 3; DC, dendritic cell; TCR, T cell receptor; TLR, Toll-like receptor. Upwards arrows mean increase/upregulation, downwards arrows mean a decrease/downregulation.

Recent studies have indicated that HDL actively contribute to the immune response to bacterial infections and mediate the response to sepsis. The contribution of HDL to immune processes is shown in Figure 3.

The participation of HDL in immune responses is related to the fact that these particles serve as a reservoir for proteins and lipids with immunomodulating properties, such as acute phase proteins, proteins of the complement system, and sphingosine-1-phosphate [45]. In addition to ApoA-I, HDL particles contain major apolipoproteins, including ApoA-II, ApoC-I, ApoC-II, ApoC-III, and Apolipoprotein M (ApoM), as well as key enzymes involved in their remodeling [46]. ApoA-II may increase the monocyte response to lipopolysaccharide (LPS), thereby playing a pro-inflammatory role [47]. ApoM can bind sphingosine 1-phosphate (S1P, see below), whose concentration is drastically decreased in sepsis and inflammatory conditions [48]. ApoM limits LPS-induced acute lung injury, as well as mortality in LPS-treated mice [49]. In addition, HDL have been reported to contain ApoL-I and haptoglobin-related protein (Hpr), which are important antimicrobial proteins that provide protection from trypanosome infections [50].

The lipids contained in HDL may display anti-infectious activity. For example, gangliosides have been used in reconstituted HDL (rHDL) to bind polymeric cholera toxin, as well as to protect cells from this biological toxin [51]. Moreover, during active and chronic inflammation, HDL might act as transporters of these molecules, thus mediating immune reactions. Some authors have proposed the role of HDL as a platform for triggering immune responses [10,30].

The contribution of HDL in inflammatory reactions has been confirmed by the changes in their blood plasma concentrations and the alterations in the composition during inflammation. During the acute phase of inflammation, HDL bind acute-phase proteins, such as serum amyloid A (SAA) and ceruloplasmin. SAA is bound by the HDL_3_ subfraction [52], and the integration of SAA into HDL particles is followed by the displacement of ApoA-I from them. Thus, SAA becomes the main (up to 80%) protein compound of HDL [43], resulting in the loss of HDL’ ability to contribute to RCT [53].

The regulation of the complement system functioning serves as another important role of HDL. Proteomic analysis of HDL particles has shown the presence of complement system proteins, such as C3, C4, and C9, as well as vitronectin and clusterin. The HDL of healthy people comprise C4a, C4b, C9, and vitronectin, while the HDL of patients with coronary artery disorder are enriched in C3 and C4 [54,55]. C3 is the key protein responsible for the activation of the complement system. Interestingly, the analysis of cultured endothelial cells has shown the blockage of membrane attack complex assembly by HDL [56,57]. On the contrary, in vivo studies have revealed a negative correlation between the HDL level and the concentration of the circulating terminal complex C5b–C9 in plasma [58]. These data are in contrast to the results of proteomic analysis. These contradictions might be explained by the prevention of excessive activation of the complement system proteins due to their clearance by HDL, which also serves as their depot. Thus, HDL might be involved in the fine regulation processes of complement system activation.

Significant changes in the levels and composition of HDL have also been observed during sepsis. It has been shown that a low level of HDL-C negatively correlates with the severity of sepsis and is associated with a massive release of inflammatory mediators (i.e., tumor necrosis factor α (TNF-α), interleukin 1β (IL-1β), interleukin 6 (IL-6), and interleukin 8 (IL-8)) [59,60]. Even though inflammation is a protective response of an organism to pathogens, uncontrolled system inflammation might cause severe complications, such as disseminated intravascular coagulation (DIC) syndrome, tissue damage, and endotoxic shock [61]. HDL are able to bind and neutralize the compounds of bacterial cells, such as bacterial endotoxin (lipopolysaccharide) and lipoteichoic acid [62,63]. It has also been observed that the intravenous administration of reconstructed HDL or ApoA-I mimetics protects mice from the toxic effects of LPS, in which a two-fold increase in the HDL level was followed by a decrease in the concentration of pro-inflammatory cytokines and an enhanced survival rate [64]. It has been emphasized that the intensity of the LPS-induced damage of organs was reduced in rats treated with HDL; the levels of inflammatory mediators (TNF-α and nitrogen oxide) also reduced under these conditions [65].

Mice with a knocked out ApoA-I gene do not have HDL, resulting in a lower ability of blood serum to neutralize LPS compared to control animals [66]. Besides LPS neutralization, HDL promote the clearance of this molecule by the SR-BI receptor [67]. The protective effects of HDL against parasitic infections are mediated by apolipoprotein ApoL-I, which is bound to HDL_3_ molecules. ApoL-I is the key component of trypanosome lytic factor-1 (TLF-1), which also contains ApoA-I and haptoglobin-binding protein. TLF-1 mediates the lysis of *Trypanosoma brucei* and *Leishmania.* This factor provides humans and the majority of primates resistance to parasitic infections [68].

It has been proposed that HDL might affect immune responses by influencing cholesterol levels in the plasmatic membrane of immune cells, thereby changing their fluidity. The changes in membrane properties (more dense lipid packing) due to the increase in cholesterol concentration in membrane microdomains, called lipid rafts, affect the processes of activation of the key receptors (i.e., Toll-like receptors, T and B cell receptors, co-stimulating molecules, and major histocompatibility complex II), thus mediating adaptive and innate immune reactions [69,70,71,72,73]. It has been shown that ApoA-I prevents the development of pro-inflammatory signaling mediated by the co-stimulating molecule CD40. ApoA-I induces changes in the cholesterol level in lipid rafts via ABCA-1-dependent efflux [74]. Another in vitro study revealed that monocyte incubation with HDL or ApoA-I reduces CD11b expression and monocyte adhesion to endothelial cells [75]. Macrophage incubation with HDL is followed by an increase in the concentration of the transcription factor ATF3 (activating transcription factor 3), thereby inhibiting Toll-like receptor 2 (TLR-2)-expression. The downregulation of TLR-2 signaling suppresses the expression of pro-inflammatory cytokines (i.e., IL-6, IL-12p40, and TNF-α) mediated by TLR-2 [76,77]. This leads to macrophage polarization from the inflammatory M1 to the anti-inflammatory M2 phenotype [44]. It has also been observed that ApoA-I reduces TLR-4 migration to lipid rafts, thus preventing TLR-4-mediated activation of the pro-inflammatory transcription factor nuclear factor kappa B (NF-kB) in endothelial cells. HDL and ApoA-I also induce a decrease in the density of major histocompatibility complex II (MHC II) expression on the membrane of dendritic cells (DCs) and reduce IL-2 production by these cells, thus suppressing their ability to activate T-lymphocytes [78,79]. It has been noted that HDL inhibit inflammatory responses by reducing the secretion of the pro-inflammatory cytokine IL-12, which promotes the differentiation of T-helpers (Th) to inflammatory Th 1 type (Th1) [80].

Based on the above data, some authors believe that the primary evolutionary role of HDL particles is scavenging and their participation in the clearance of infectious material. HDL have a great influence on innate immunity, and these data may explain their great impact on atherosclerosis, because it is well accepted that chronic inflammation is one of the driving forces of atherogenesis.

#### 3.2.2. Antioxidant Properties

Due to their antioxidant properties, HDL can protect LDLs against oxidation by reactive oxygen species (ROS). HDL contain enzymes with antioxidant activities, such as paraoxonase 1 (PON1), as well as other antioxidants, particularly carotenoids and vitamin E [81,82,83,84]. The antioxidant potential of HDL has been shown to be impaired in patients with dyslipidemia [85].

#### 3.2.3. Anti-Inflammatory Properties

The anti-inflammatory properties of native HDL allow them to inhibit vessel inflammation. These biological effects of HDL are mediated by the suppression of pro-inflammatory responses mediated by cell nuclear factor NF-κB, as well as a reduction in the expression of adhesion molecules (i.e., vascular cell adhesion molecule 1 (VCAM-1) and E selectin) [81] and the production of chemoattractants of blood monocytes IL-8 and monocyte chemoattractant protein 1 (MCP-1) [85].

HDL have been reported to limit inflammasome activation by cholesterol crystals by reducing NRF3 (NLR Family Pyrin Domain Containing 3) expression [86]. In patients with acute coronary syndrome, the anti-inflammatory properties of HDL are shifted toward pro-inflammatory properties due to the oxidation of particles [87].

#### 3.2.4. Vasodilating and Endothelial-Protecting Properties

HDL possess vasodilating properties due to endothelial NO-synthase (eNOS) activation [43]. Sphingosine-1-phosphate (S1P) and SR-BI receptor also contribute to these effects of HDL. S1P is transported by HDL and plays a crucial role in the maintenance of the normal functioning of vessels [43]. In vitro studies have shown that S1P transport is mediated by ApoM-containing HDL particles. The interaction between S1P and its receptor on endothelial cells results in the activation of Akt kinase (RAC-alpha serine/threonine-protein kinase, Protein kinase B alpha) and eNOS, which determines the maintenance of endothelial barrier integrity [88,89]. Interestingly, the S1P level significantly decreases during the acute phase of inflammation. S1P directly affects macrophages, which express specific S1P receptors, particularly S1PR1 and S1PR2 receptors, on membranes. It has been shown that S1P inhibits LPS-induced production of pro-inflammatory cytokines by macrophages and their recruitment to inflammation loci.

It has also been observed that the protective effects of HDL under normal conditions depend on the activity of PON1. The reduced activity of PON1 in patients with cardiovascular disorders suppresses the vasoactive properties of HDL [81].

#### 3.2.5. Antithrombotic Properties

The antithrombotic effects of HDL are mediated by the suppression of the expression of the tissue factor and activation factor X, as well as the secretion of the inhibitor of the plasminogen activator [90]. HDL block the production of thromboxane A 2 and the synthesis of platelet-activating factor (PAF), resulting in the inhibition of thrombocyte aggregation [91]. HDL levels are decreased in patients with venous thrombosis.

In addition to their direct action on coagulation, HDL may also limit the prothrombotic effect of overactivated neutrophils. In response to LPS, neutrophils have been shown to expel their DNA, forming a net that traps bactericidal proteins, such as elastase, cathepsin G, or histones, aimed at limiting the spreading of bacteria. These NETs (or neutrophil extracellular traps) also promote the formation of a thrombus in collaboration with platelets via their TLR-4 to confine bacteria [46].

#### 3.2.6. Contribution of HDL to MicroRNA Transport

MicroRNAs (miRNAs) are short non-coding regulatory RNAs, 18–25 (average of 22) nucleotides in length. They contribute to the transcriptional and posttranscriptional regulation of gene expression via RNA interference. miRNAs were first observed in *Caenorhabditis elegans* [92] and afterward found in the majority of eukaryotes [93]. It has been proposed that miRNA sequences compose 1%–5% of the human genome and regulate up to 30% of protein-coding genes [94]. Nowadays, more than a thousand human miRNAs have been discovered [95] in practically all organs and tissues of an organism. Besides intracellular miRNAs, extracellular (circulating) miRNAs have been registered [96]. Circulating miRNAs are transported from one cell (donor) to another (recipient) within exosomes, apoptotic bodies, microvesicles, ribonucleoproteins (nucleophosmin 1 and argonaut 2), and, as recently shown, lipoproteins.

The hypothesis regarding the possible transport of endogenous miRNAs by HDL was first proposed by Vickers et al. [97]. They isolated and characterized the complexes of miRNAs and HDL in healthy volunteers and patients with hereditary dyslipidemia [97]. Significant differences in the levels of certain types of miRNAs in the HDL of healthy volunteers and patients with impaired lipid metabolism were observed. For example, hsa-miR-223, hsa-miR-105, and hsa-miR-106a were observed in the HDL of patients with familial hypercholesterolemia. On the contrary, the HDL of healthy volunteers mostly contained miR-135a, hsa-miR-188-5p, and hsa-miR-877. Moreover, the transport of these miRNAs by HDL suppresses the functioning of corresponding mRNA targets in cultivated hepatocytes, reflecting the functional activity and importance of complexes of HDL and miRNAs. LDLs have also been shown to bind miRNAs, but their composition differs from miRNAs bound to HDL. Distinct from exosomes and microvesicles containing both miRNAs and fragments of mRNAs, highly purified HDL transport only miRNAs of 22–26 nucleotides in length [97].

In vitro and in vivo experiments have shown that miRNAs might effectively bind to native and reconstructed HDL particles and be transported to the liver [98]. It has been observed that the transport of a complex of miR-223 and HDL to the endothelial cells provides a significantly higher endogenous concentration of miR-223 and suppresses the expression of intercellular adhesion molecule 1 (ICAM-1), thus mediating the pro-inflammatory effects. Interestingly, miR-223 is not expressed in endothelial cells. Moreover, the HDL of mice with a knocked out *MIR-223* gene do not possess these effects [99].

The study by Niculescu et al. showed that the profile of miRNAs bound to HDL isolated from patients with stable coronary artery disease differs from that of other groups of patients [100]. The authors revealed significant differences in the concentrations of hsa-miR-486 and hsa-miR-92a in patients of at-risk groups and patients with stable coronary artery disease [100]. HDL might also contain miRNAs that mediate angiogenesis and inflammation, such as hsa-miR-92a, hsa-miR-126, hsa-miR-150, and hsa-miR-378 [101]. The concentration of the pro-inflammatory miRNA hsa-miR-155, which is specifically expressed in macrophages of atherosclerotic plagues, is significantly increased in patients with coronary syndrome [101].

The mechanisms of the loading of HDL particles with miRNAs remain to be elucidated. The transport of miRNAs to HDL is suppressed by the ceramide signaling pathway, mediating the miRNA export from cells by exosomes [102]. In turn, miRNA delivery directly to the target cells might be mediated by ABCA1 transporters and SR-B1 receptors [97].

Therefore, the literature data indicate the possibility of using certain types of miRNAs and their profiles as biomarkers of cardiological and metabolic disorders. The contribution of HDL to the mechanisms of intracellular interactions might uncover new functions of these ancient molecules of plasma. Thus, new questions on the mechanisms of the biological functions of these molecules arise, and new fundamental investigations of these processes are required.

## 4. Conclusions

Herein, we summarized the recent literature data on the structure and pleiotropic functions of HDL in the context of maintaining the homeostasis of blood plasma and, therefore, the whole body. Like other scientists, we propose that HDL have an important evolutionary role and serve as an ancient platform for maintaining many aspects of an organism’s homeostasis.

The majority of relevant studies have indicated that the classical understanding of HDL as cholesterol transporters from tissues to the liver should be expanded.

HDL are complex natural nanoparticles that undergo dynamic remodeling through interactions with various enzymes and tissues throughout their life cycle. The presence of many proteins in the composition of HDL might be temporary and related to certain physiological processes in organisms. These data confirm the hypothesis that HDL are dynamic multifunctional structures that serve as an integral platform mediating several physiological functions. HDL display important functions in many aspects of immunity, tissue homeostasis, and intercellular signaling in invertebrates and vertebrates and can be treated as homeostatic particles of blood plasma.

The new data on the role of HDL in miRNA transport indicate an important role of HDL in intracellular interactions. The multifunctionality and high heterogeneity of HDL reflect the necessity of further investigations of the structural and functional features of HDL under physiological and pathological conditions. In particular, the possibility of using HDL molecules as a delivery system is of specific interest. Reconstituted HDL are biomimetic nanoparticles, which can be used for the safe delivery of gene-therapeutic substances to cells for the thin manipulation of genetic impairments during atherosclerosis and other diseases.

The accumulated data on HDL functions open up new possibilities for obtaining a deeper understanding of the pathogenesis of certain disorders, cell-to-cell interactions, and immune reactions. These data are important for the development of a new class of biomimetic medicinal substances based on natural nanoparticles, such as HDL.

## Figures and Tables

**Table 1:** Main subclasses of high-density lipoproteins (HDL) (adapted from [18]).

Density, g/mL	HDL Separation by Ultracentrifugation		HDL Fractions Separation by Electrophoresis	
	HDL Fractions	Diameter, nm	HDL Subfractions	Diameter, nm
1.063–1.125	HDL_2_	8.8–12	HDL_2b_ *	9.7–12
			HDL_2a_ *	8.8–9.7
1.125–1.21	HDL_3_	7.2–8.8	HDL_3a_ *	8.2–8.8
			HDL_3b_ *	7.8–8.2
			HDL_3c_ *	7.2–7.8
>1.21	preβ-HDL		preβ-HDL **	

* Gradient non-denaturing polyacrylamide gel electrophoresis (GGE); ** agarose gel, two-dimensional gel electrophoresis (2-DE).

**Table 2:** Basic HDL proteins and their functions (adapted from [18]).

Protein	Origin and Biological Function
ApoA-I	The main structural and functional apolipoprotein, which interacts with cellular receptors, activates lecithin–cholesterol acyltransferase (LCAT) and exhibits antiatherogenic activity. The main sites for ApoAI synthesis and secretion are the liver and small intestine.
ApoA-II	Structural and functional apolipoprotein, predominantly synthesized in the liver.
ApoA-IV	Structural and functional apolipoprotein, synthesized in the intestine.
ApoC-I	Possesses a high positive charge and, thus, can bind free fatty acids, can modulate the activity of some of the proteins involved in HDL metabolism, can activate LCAT, and can inhibit hepatic lipase and cholesterol ester transport protein (CETP).
ApoC-II	Activates lipoprotein lipase (LPL).
ApoC-III	LPL and hepatic lipase inhibitor.
ApoC-IV	Regulator of triglyceride (TG) metabolism.
ApoD	Responsible for the binding and transport of small hydrophobic molecules. Expressed in many tissues, including the liver and the intestines.
ApoE	Structural and functional apolipoprotein, a ligand for low-density lipoprotein (LDL) receptors and LDL receptor-associated protein (LRP), and binds to glycosaminoglycans on cells. Synthesized in several tissues and cell types, including the liver, endocrine tissues, central nervous system, and macrophages.
ApoF	Inhibitor of cholesterol ester transport protein (CETP). It is synthesized in the liver.
ApoH	Binds negatively-charged molecules, primarily cardiolipin, and prevents the activation of the blood coagulation cascade by binding to phospholipids on the surface of damaged cells. Regulates platelet aggregation and is expressed in the liver.
ApoJ	Binds hydrophobic molecules and interacts with cell receptors
ApoL-I	The main component of the serum trypanolytic factor. It is expressed in the pancreas, lungs, prostate, liver, placenta, and spleen.
ApoM	Binds small hydrophobic molecules, primarily sphingosine-1-phosphate (S1P), as well as oxidized phospholipids. It is synthesized in the liver and kidneys.
PON1 (paraoxonase 1)	Ca^2+^- dependent lactonase with antioxidant properties, mainly synthesized in the liver, but also in the kidneys and colon.

**Table 3:** Proteins associated with HDL and their functions (adapted from [18]).

Protein	Biological Function
Enzymes	
LCAT (lecithin–cholesterol acyltransferase)	Esterifies cholesterol to cholesterol esters. LCAT is mainly expressed in the liver and, to a lesser extent, in the brain and testes.
PAF-AH (platelet-activating factor acetyl hydrolase; lipoprotein-associated phospholipase A2 (LpPLA2))	Hydrolyzes short-chain oxidized phospholipids. Synthesized in the brain, white adipose tissue, and placenta. Macrophages are the most important source of the circulating enzyme.
GSPx-3 (glutathione selenoperoxidase 3)	A component of the system of protection against the oxidative damage of molecules. Catalyzes the redox reaction of peroxides (hydrogen peroxide to water or lipid peroxides to the corresponding alcohols) with glutathione. It is synthesized in the liver, kidneys, heart, lungs, mammary glands, and placenta.
Lipid transport proteins	
PLTP (phospholipid transfer protein)	Remodels HDL into large and small particles and binds and transports bacterial lipopolysaccharide. It is synthesized in the placenta, pancreas, lungs, kidneys, heart, liver, skeletal muscles, and brain. It is also a positive marker of the acute phase of inflammation.
CETP (cholesterol ester transport protein)	Provides heteroexchange of cholesteryl ester (CE) and TG and homoexchange of phospholipid (PL) between HDL and ApoB-containing lipoproteins. It is synthesized in the liver and adipose tissue.
Acute-phase proteins	
SAA1 (serum amyloid A1)	Major acute-phase reactant. Formed preferably in the liver.
SAA4 (serum amyloid A4)	Minor acute-phase reactant. Formed preferably in the liver.
Alpha-2-HS glycoprotein	Negative acute-phase reactant, which promotes endocytosis and opsonization. It is synthesized in the liver.
Fibrinogen alpha chain	Fibrin precursor, main component of blood clots and platelet aggregation.
Complement system proteins	
C3	One of the main activators of the complement system through classical and alternative paths.
Proteinase inhibitors	
α-1-antitrypsin	Inhibits serine proteases, especially neutrophil elastase.
Hrp (haptoglobin-related protein)	Decoy substrate to prevent proteolysis.
Other proteins	
Transthyretin	Thyroid hormone binding and transport.
Serotransferin	Iron binding and transport.
Vitamin D-binding protein	Vitamin D binding and transport.
α-1B-glycoprotein	Unknown.
Hemopexin	Heme binding and transport.

## Abbreviations

ABCA1	ATP Binding Cassette Subfamily A Member 1
ABCG1	ATP Binding Cassette Subfamily G Member 1
CETP	cholesteryl ester transfer protein
DC	dendritic cell
GGE	gradient non-denaturing polyacrylamide gel electrophoresis
GSPx-3	glutathione selenoperoxidase 3
HDL	high-density lipoproteins
Hrp	haptoglobin-related protein
ICAM-I	intercellular adhesion molecule 1
IL	interleukin
LCAT	lecithin/cholesterol acyltransferase
LDL	low-density lipoproteins
LDLR	low-density lipoprotein receptor
LP	lipoprotein
LPL	lipoprotein lipase
LpPLA2	lipoprotein-associated phospholipase A2
LRP	LDL receptor-related protein
LPS	lipopolysaccharide
NETs	neutrophil extracellular traps
NF-kB	nuclear factor kappa B
NRF3	NLR Family Pyrin Domain Containing 3
MHC	major histocompatibility complex
PAF-AH	platelet-activating factor acetyl hydrolase
PL	phospholipid
PLTP	phospholipid transfer protein
PON1	paraoxonase 1
SAA	serum amyloid A
S1P	sphingosine-1-phosphate
SR-BI	scavenger receptor class B type I
TG	triglyceride
TLF-1	trypanosome lytic factor-1
TLR	Toll-like receptor
TNF-α	tumor necrosis factor α
TCR	T cell receptor
VCAM-1	vascular cell adhesion molecule 1
VLDL	very-low-density lipoproteins
